# Modeling the dynamics of neural codes in the olfaction of the *Manduca-sexta* moth

**DOI:** 10.1186/1471-2202-13-S1-O18

**Published:** 2012-07-16

**Authors:** Eli Shlizerman, Jeffrey Riffell, J Nathan Kutz

**Affiliations:** 1 Department of Applied Mathematics, University of Washington, Seattle, WA 98195, USA; 2 Department of Biology, University of Washington, Seattle, WA 98195, USA

## 

The Anntenal Lobe (AL) is the olfactory processing unit in insects, composed of projection neurons (PNs) and local neurons (LNs) [1]. It has been demonstrated that the AL reformats the sensory input information that it receives into spatiotemporal firing patterns exhibited by the PNs [2,3]. In many insects the LNs are mainly inhibitory which suggests that the inhibition is responsible for shaping the input into a robust pattern [4]. The robustness of the pattern is expressed as follows: over several applications of the same odor the projection of the data recorded from the PNs onto a few dominant firing patterns results in a robust low dimensional trajectory. This trajectory appears to be similar to a trajectory that converges to a stable unique fixed point [5].

In this work, we resolve several open questions raised by [5,6]. Specifically, we study how interactions between the LNs and PNs permit creation of robust and spatiotemporal codes. We further propose a simple model that mimics the dynamics of the AL and the dynamics of mixtures of odors. Our work is a combination of theoretical analysis and experimental studies.

Theoretically we consider a network of *excitatory-inhibitory firing units* that has a similar structure to the AL. By proposing to *project the dynamical equations* of the network onto given orthogonal spatial patterns, we derive the conditions on the interactions between the inhibitory and excitatory population such that the network will support a unique stable fixed point. The analysis is based on requiring that the inhibitory neurons will suppress inputs that are not associated to specific odors (noise and unknown patterns) but being neutral to the given spatial patterns. These conditions allow us to *prescribe* the connections between PNs and LNs and do not require specific symmetries (in contrast to Hopfield networks).

Experimentally we record the dynamics of PNs in the AL of the Manduca Sexta moth both for inputs that are single odor or mixtures of two odors. Extracting the spatial patterns (first PCA mode) obtained from experiments with single odor inputs we calibrate the network. We are able to establish *similarity between the model dynamics and experimental projections* and thus validate our theoretical construction. Once the model is calibrated we test it against experiments with inputs that are different ratios of two odors revealing similar dynamics. To test the importance of inhibition, conjectured to be responsible for existence of a stable fixed point, we repeat the experiments in which the *inhibition is blocked by a drug*. Results demonstrate that the robustness of the dynamics is destroyed.
